# Miscellany

**Published:** 1902-08

**Authors:** 


### Miscellany.

Arsenical Poisoning cured by Orthoform.—I had a case of acute arsenical poisoning with the conditions present so familiar to all. With a spoon excavator I removed the blackened gum tissue, syringed the parts with a warm antiseptic solution, soaked the gums and surrounding tissues with dialyzed iron, and applied a twenty-five per cent. unguent orthoform. The pain in this case was the severest that I ever saw, and I must admit being astonished when the patient returned on the following day and reported that the pain had ceased and had not returned. Relief came within twenty minutes.—A. D. Kyner, *Items of Interest.*

---

Die and Counter-Die of Mellotte's Metal.—Dr. A. H. Davis, in the Atlanta *Dental Journal,* recommends painting the surface of the die with a thin coat of whiting and water to prevent adhesion. It is then dried with a blast from the blow-pipe. The counter-die may be poured reasonably hot. The parts separate readily.

[If No. 4 tin-foil be pasted over the die and pressed closely in contact with a soft piece of rubber and then dried, the die and counter-die will separate readily. If another coat of the paste be put over the tin next to the counter-die, the tin will not adhere to the counter-die. If this be not done the composition of the metal will be gradually changed by the addition of the tin.

The paste used is ordinary paste sold at stationery stores.—McClain.]

---

Burlesque Advertising as a Means of exposing Fakirs.—The dental parlors at Altoona, Pa., became so extravagant recently in their promises and advertisements that several of the reputable dentists of the city issued an unsigned and clever burlesque advertisement, which it is said had a wholesome effect in enlightening the community as to the methods and promises of fakirs.—*Dental Digest.*

A Method of making Eucalypto-Percha.—Dr. W. F. Green first dissolves gutta-percha in chloroform, allowing it to stand a sufficient time to become thoroughly dissolved. Eucalyptus oil is then added to the chloropercha, and the chloroform is allowed to evaporate through antiseptic gauze. More eucalyptus oil is gradually added until the mass is of a proper consistency. While this method is not new, he claims that by so doing he gets the gutta-percha into a more finely comminuted form than could be done by the simple use of eucalyptus oil. By the aid of the microscope the mass to all appearances is much more homogeneous than is chloro-percha.—*Review.*

---

How to splice Engine Bands.—The manner in which most dentists splice their bands is, to say the least, a very clumsy one. It takes considerable time to make it, it is not very strong, and never runs smoothly. The splice which I recommend is made very quickly, makes a strong even splice, and runs smoothly; also the harder you pull on the band the stronger it holds. The instrument, which I shall call a needle, used in making the splice is made of piano wire, bent in the form of a hair-pin. The free ends are inserted in a wooden handle and fastened so that they will not pull out, allowing the bow end to extend about two and one-half inches from the handle. The sides of the bow must be bent near enough to allow it to pass easily through the centre of the band.

To make the splice, measure the exact length the band must be when spliced, mark it, then cut off the band, say, seven inches longer. This extra length is taken up in the splice. A splice six inches long is stronger and runs smoother than one four inches long. Unravel about an inch of each end of the band. Take the needle and pass the bow into the band where you marked the end to be, then pass it through the band one-half of the extra length and then out again. Take the other end of the band and insert into the bow of the needle just enough to hold, and pull it through and out where the needle first entered. Treat the other end of the band in the same way as the first and draw the free end through. Smooth out the splice, and cut the ends so that they will come inside the band, and your splice is finished.

If you wish to make the splice smoother, roll it between two pieces of wood.

If the band has a core, it is more painstaking to make the splice, yet it is easily done. First draw out the core from each end the length the splice is to be, say six inches, and so manipulate it as to have the ends come inside the band exactly where the core has been cut.

If you are not particular about this, there will be a weak spot at each end of the splice. With pains in the making you will hardly be able to detect the splice after it is finished.—GEO. A. MAXFIELD, *Catching's Compendium.*

